# Supplementary material for: Improved Anti-Biofilm Effect against the Oral Cariogenic Streptococcus mutans by Combined Triclosan/CBD Treatment
Source: Biomedicines. 2023 Feb 10;11(2):521. doi: 10.3390/biomedicines11020521 (PMC9953046; doi:10.3390/biomedicines11020521)
Supplement: Supplementary file 1 [file biomedicines-11-00521-s001.zip › biomedicines-2189595-supplementary.pptx]

## Slide 1
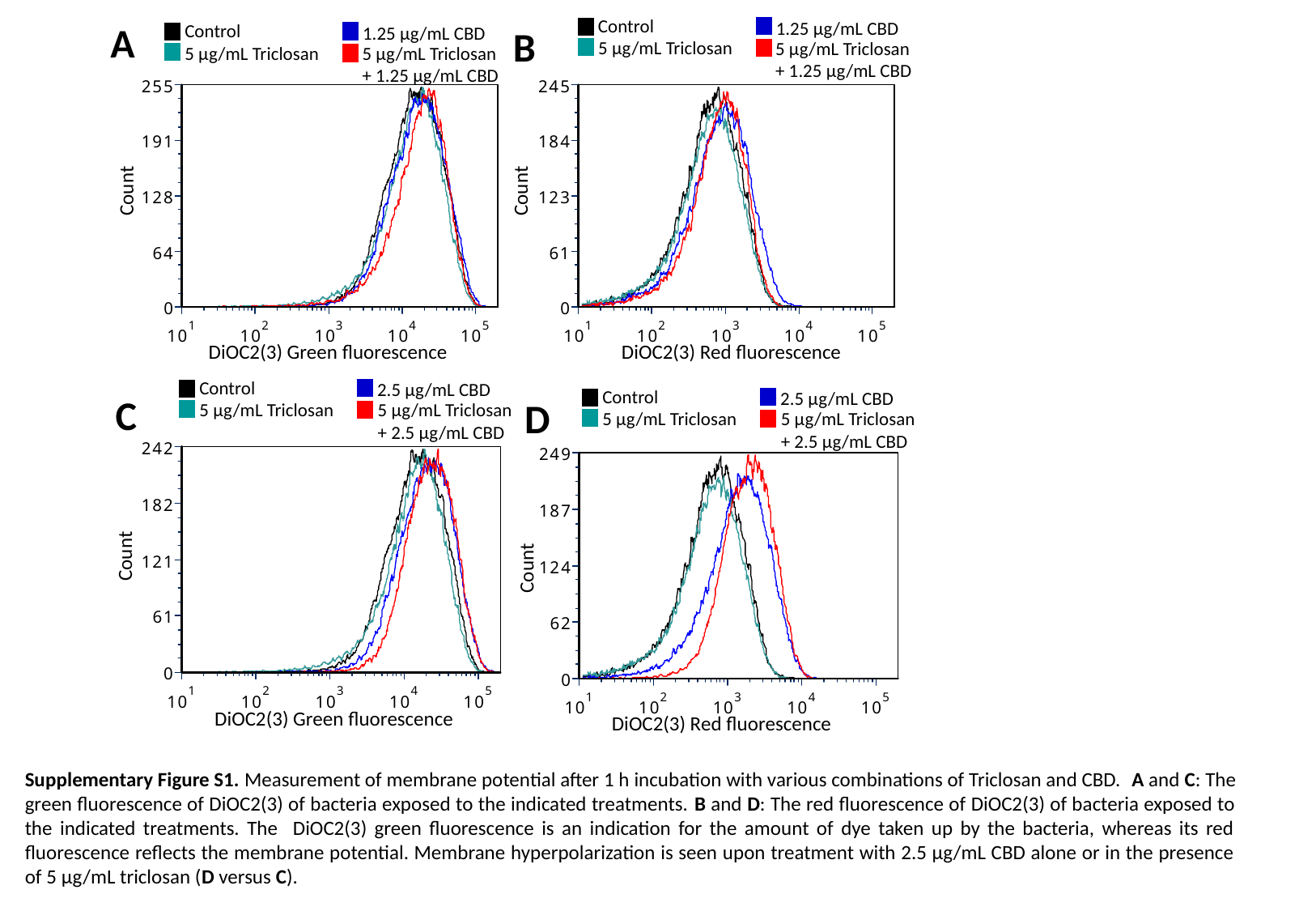

Control
5 µg/mL Triclosan
1.25 µg/mL CBD
5 µg/mL Triclosan
+ 1.25 µg/mL CBD
A
Control
5 µg/mL Triclosan
1.25 µg/mL CBD
5 µg/mL Triclosan
+ 1.25 µg/mL CBD
B
Count
Count
DiOC2(3) Red fluorescence
DiOC2(3) Green fluorescence
Control
5 µg/mL Triclosan
2.5 µg/mL CBD
5 µg/mL Triclosan
+ 2.5 µg/mL CBD
Control
5 µg/mL Triclosan
2.5 µg/mL CBD
5 µg/mL Triclosan
+ 2.5 µg/mL CBD
C
D
Count
Count
DiOC2(3) Green fluorescence
DiOC2(3) Red fluorescence
Supplementary Figure S1. Measurement of membrane potential after 1 h incubation with various combinations of Triclosan and CBD. A and C: The green fluorescence of DiOC2(3) of bacteria exposed to the indicated treatments. B and D: The red fluorescence of DiOC2(3) of bacteria exposed to the indicated treatments. The DiOC2(3) green fluorescence is an indication for the amount of dye taken up by the bacteria, whereas its red fluorescence reflects the membrane potential. Membrane hyperpolarization is seen upon treatment with 2.5 µg/mL CBD alone or in the presence of 5 µg/mL triclosan (D versus C).

## Slide 2
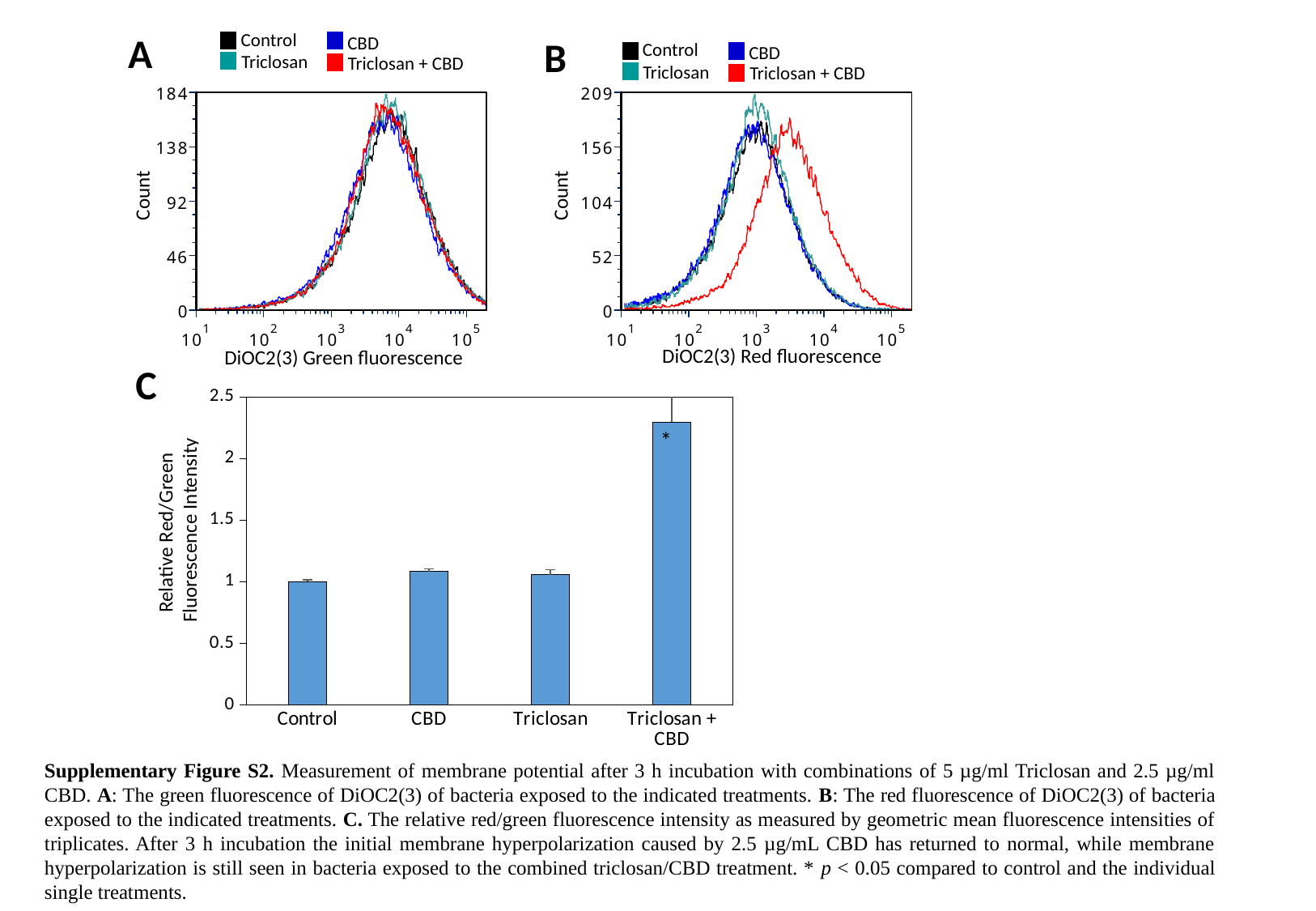

A
Control
Triclosan
CBD
Triclosan + CBD
B
Control
Triclosan
CBD
Triclosan + CBD
Count
Count
DiOC2(3) Red fluorescence
DiOC2(3) Green fluorescence
C
### Chart
| Category | |
|---|---|
| Control | 1.0 |
| CBD | 1.0840371098418986 |
| Triclosan | 1.057763063675289 |
| Triclosan + CBD | 2.296825804613352 |*
Relative Red/Green
 Fluorescence Intensity
Supplementary Figure S2. Measurement of membrane potential after 3 h incubation with combinations of 5 µg/ml Triclosan and 2.5 µg/ml CBD. A: The green fluorescence of DiOC2(3) of bacteria exposed to the indicated treatments. B: The red fluorescence of DiOC2(3) of bacteria exposed to the indicated treatments. C. The relative red/green fluorescence intensity as measured by geometric mean fluorescence intensities of triplicates. After 3 h incubation the initial membrane hyperpolarization caused by 2.5 µg/mL CBD has returned to normal, while membrane hyperpolarization is still seen in bacteria exposed to the combined triclosan/CBD treatment. * p < 0.05 compared to control and the individual single treatments.

## Slide 3
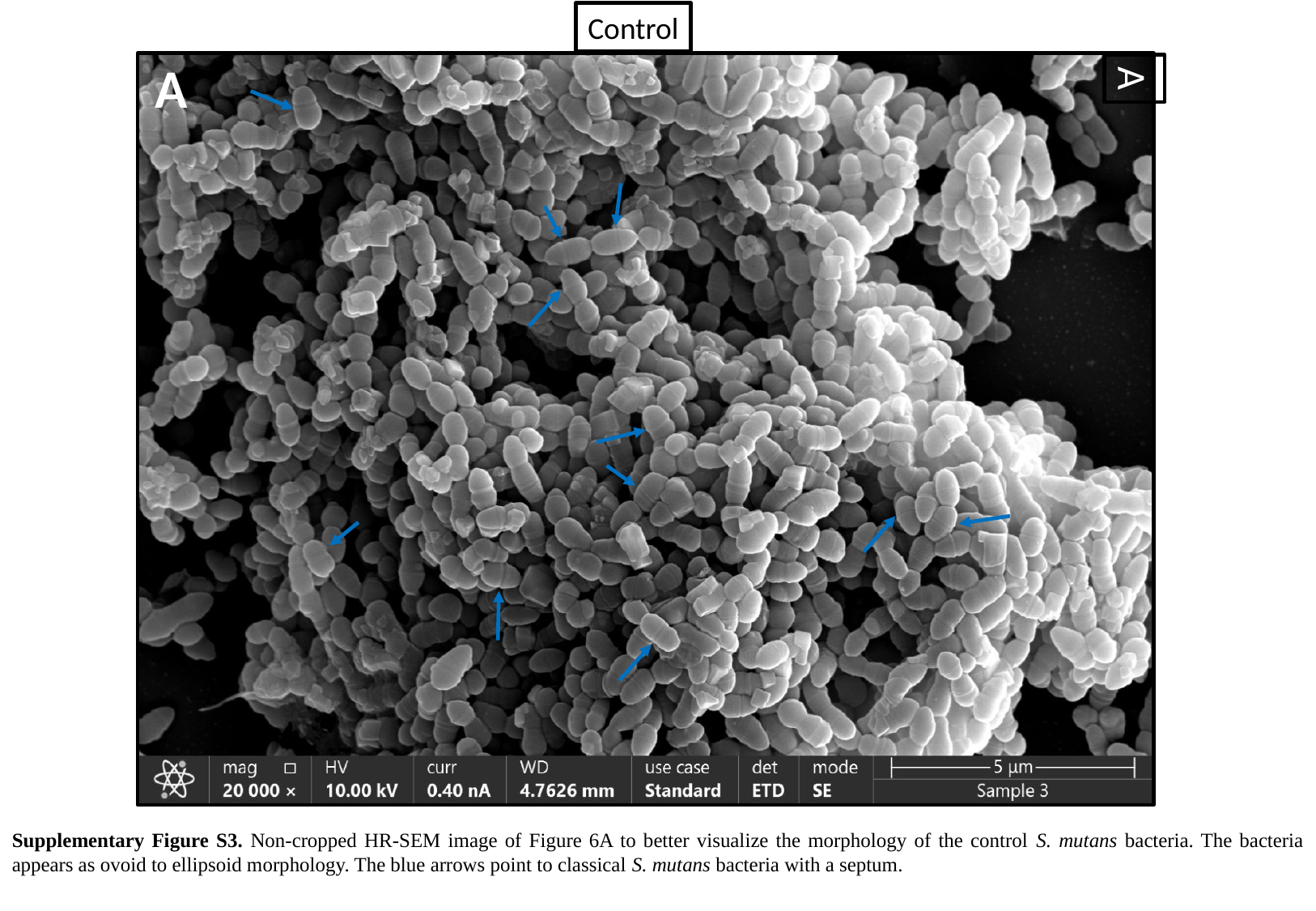

Control
A
A
Supplementary Figure S3. Non-cropped HR-SEM image of Figure 6A to better visualize the morphology of the control S. mutans bacteria. The bacteria appears as ovoid to ellipsoid morphology. The blue arrows point to classical S. mutans bacteria with a septum.

## Slide 4
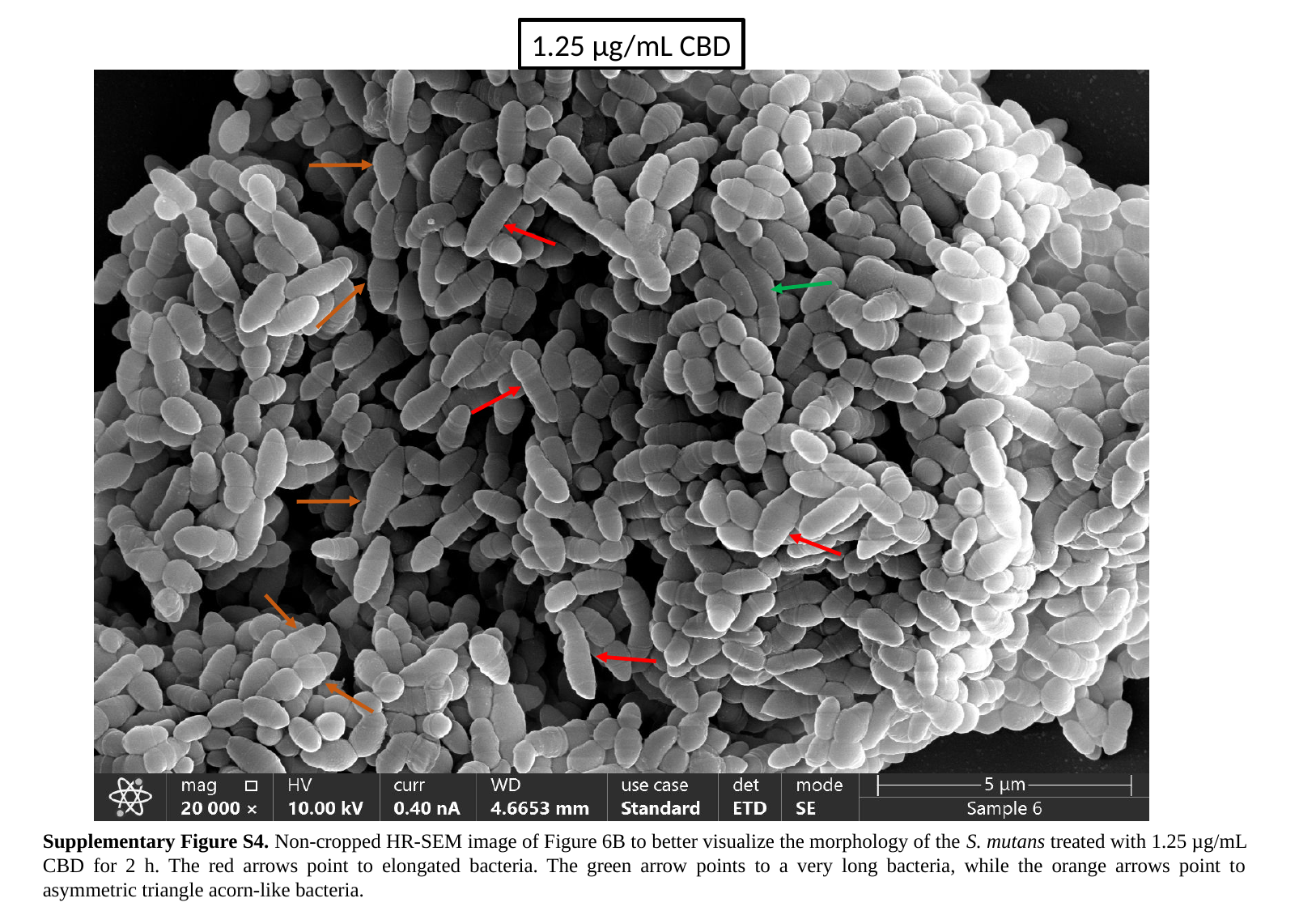

1.25 µg/mL CBD
Supplementary Figure S4. Non-cropped HR-SEM image of Figure 6B to better visualize the morphology of the S. mutans treated with 1.25 µg/mL CBD for 2 h. The red arrows point to elongated bacteria. The green arrow points to a very long bacteria, while the orange arrows point to asymmetric triangle acorn-like bacteria.

## Slide 5
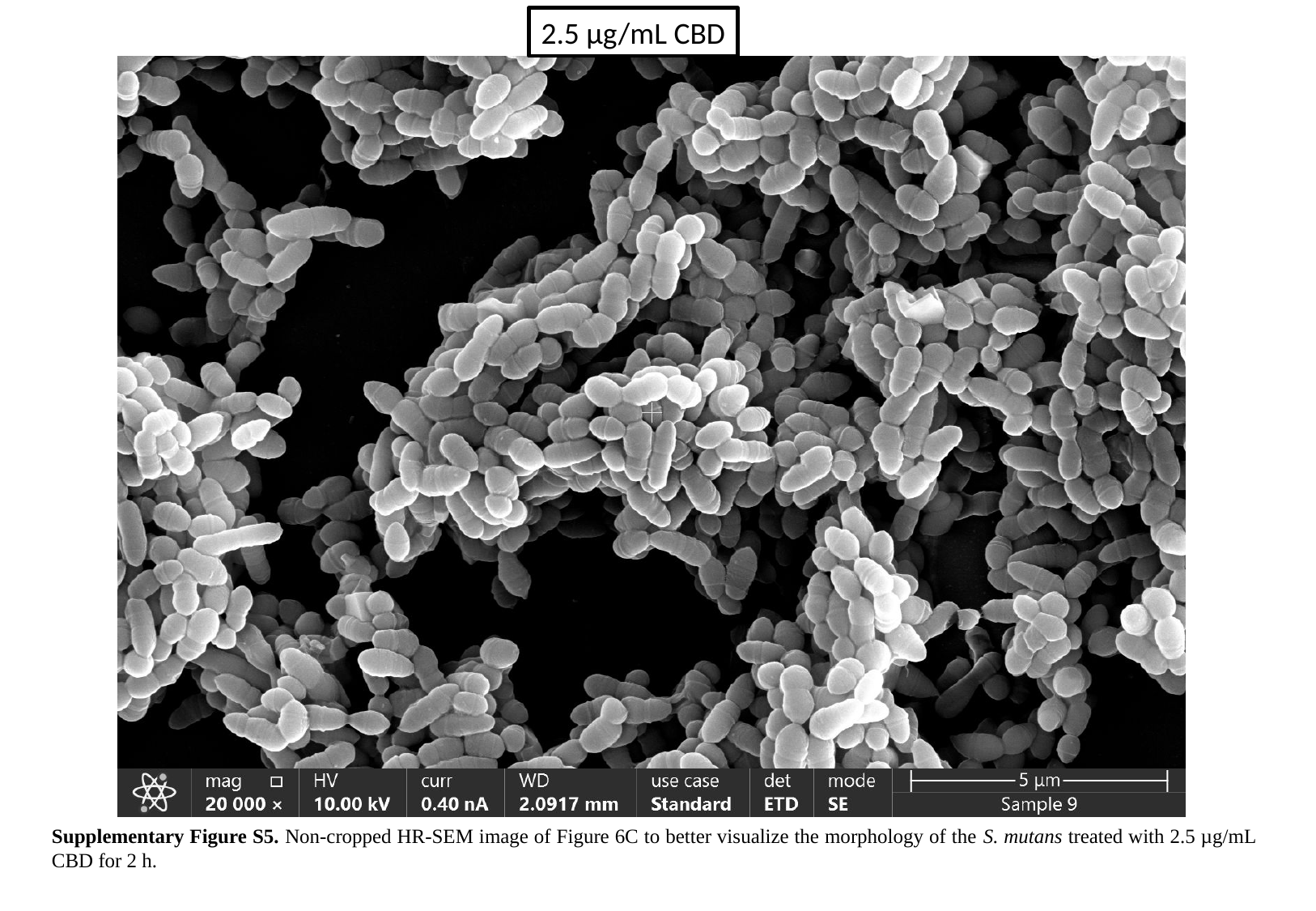

2.5 µg/mL CBD
Supplementary Figure S5. Non-cropped HR-SEM image of Figure 6C to better visualize the morphology of the S. mutans treated with 2.5 µg/mL CBD for 2 h.

## Slide 6
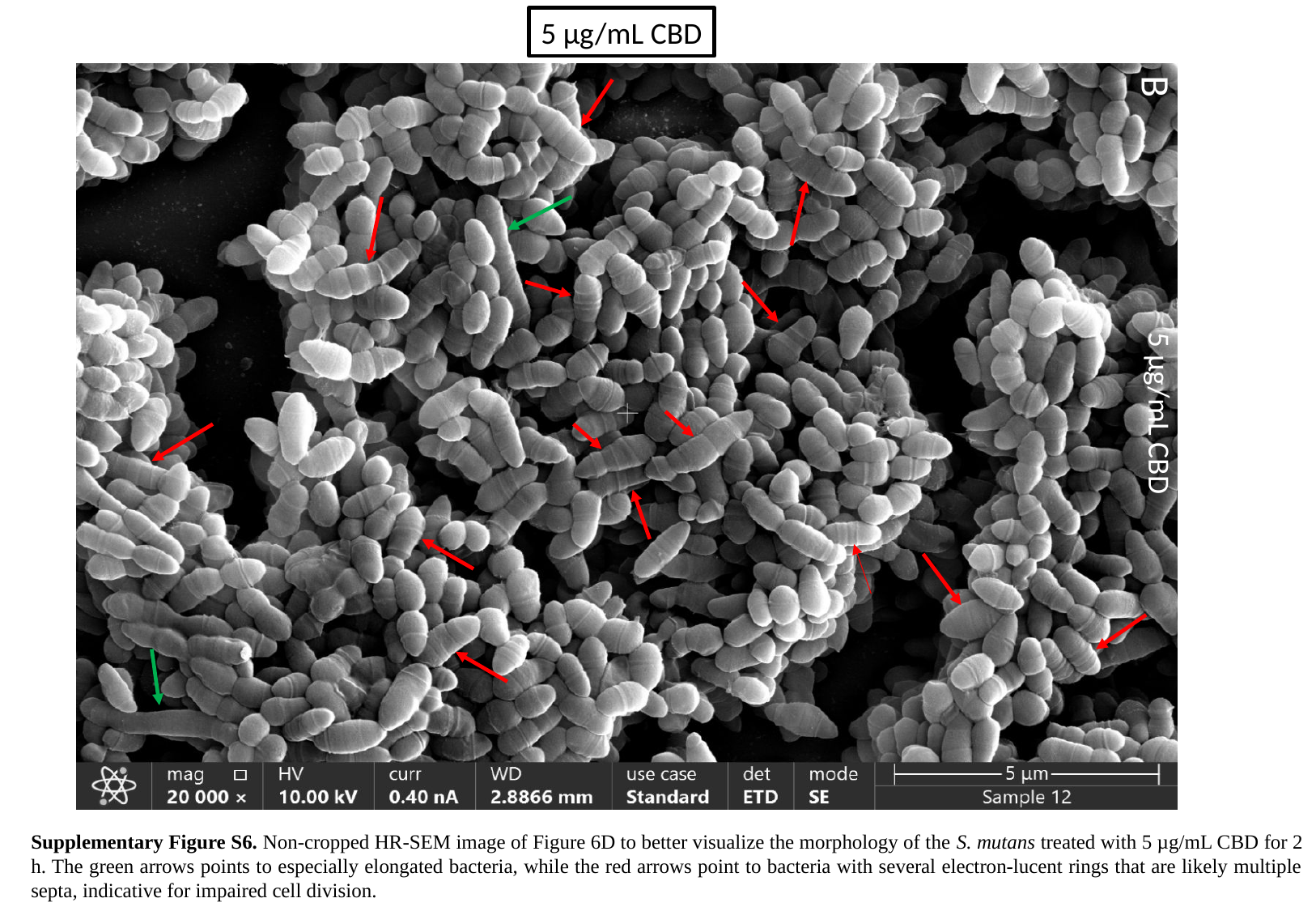

B
5 µg/mL CBD
5 µg/mL CBD
Supplementary Figure S6. Non-cropped HR-SEM image of Figure 6D to better visualize the morphology of the S. mutans treated with 5 µg/mL CBD for 2 h. The green arrows points to especially elongated bacteria, while the red arrows point to bacteria with several electron-lucent rings that are likely multiple septa, indicative for impaired cell division.

## Slide 7
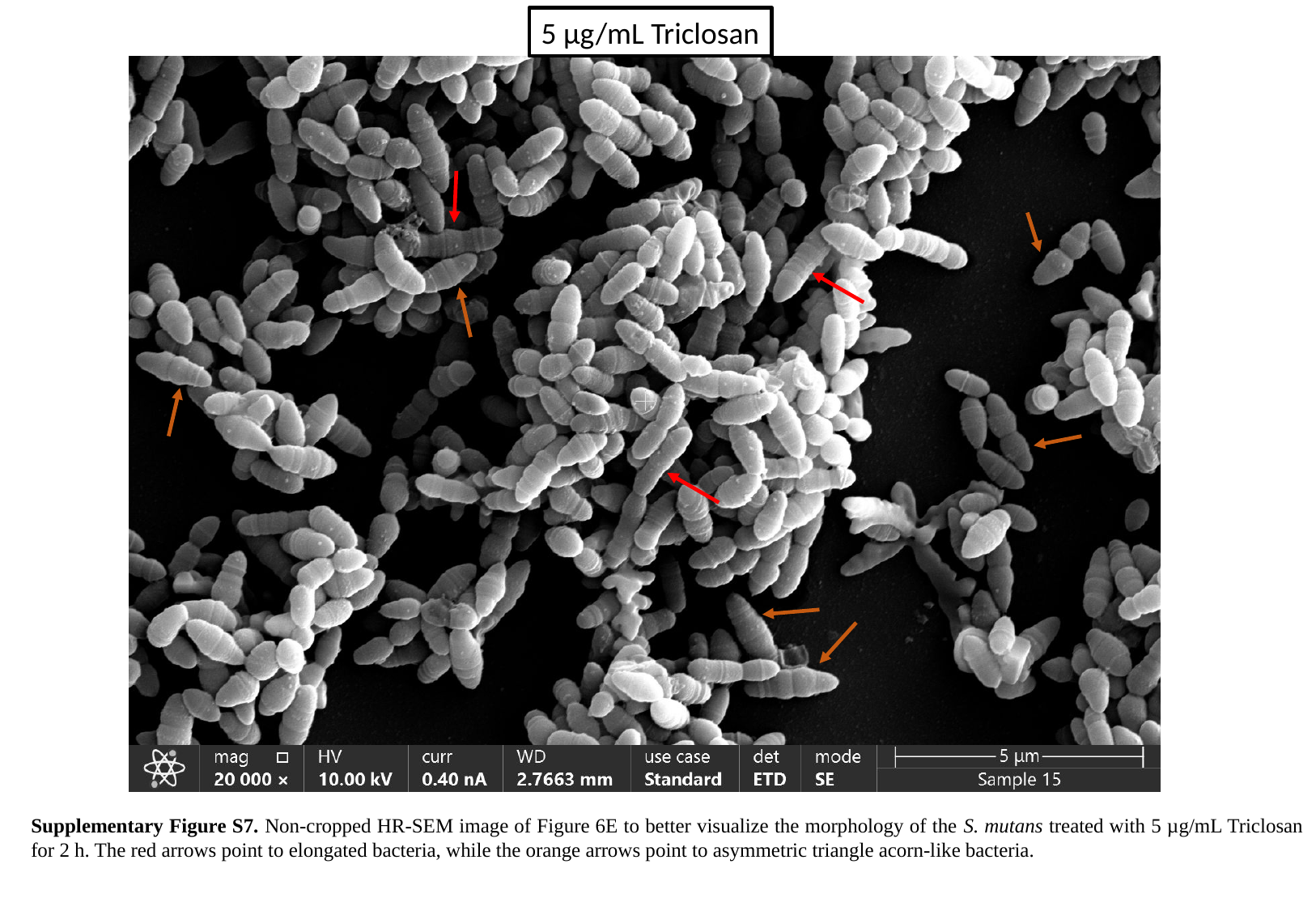

5 µg/mL Triclosan
Supplementary Figure S7. Non-cropped HR-SEM image of Figure 6E to better visualize the morphology of the S. mutans treated with 5 µg/mL Triclosan for 2 h. The red arrows point to elongated bacteria, while the orange arrows point to asymmetric triangle acorn-like bacteria.

## Slide 8
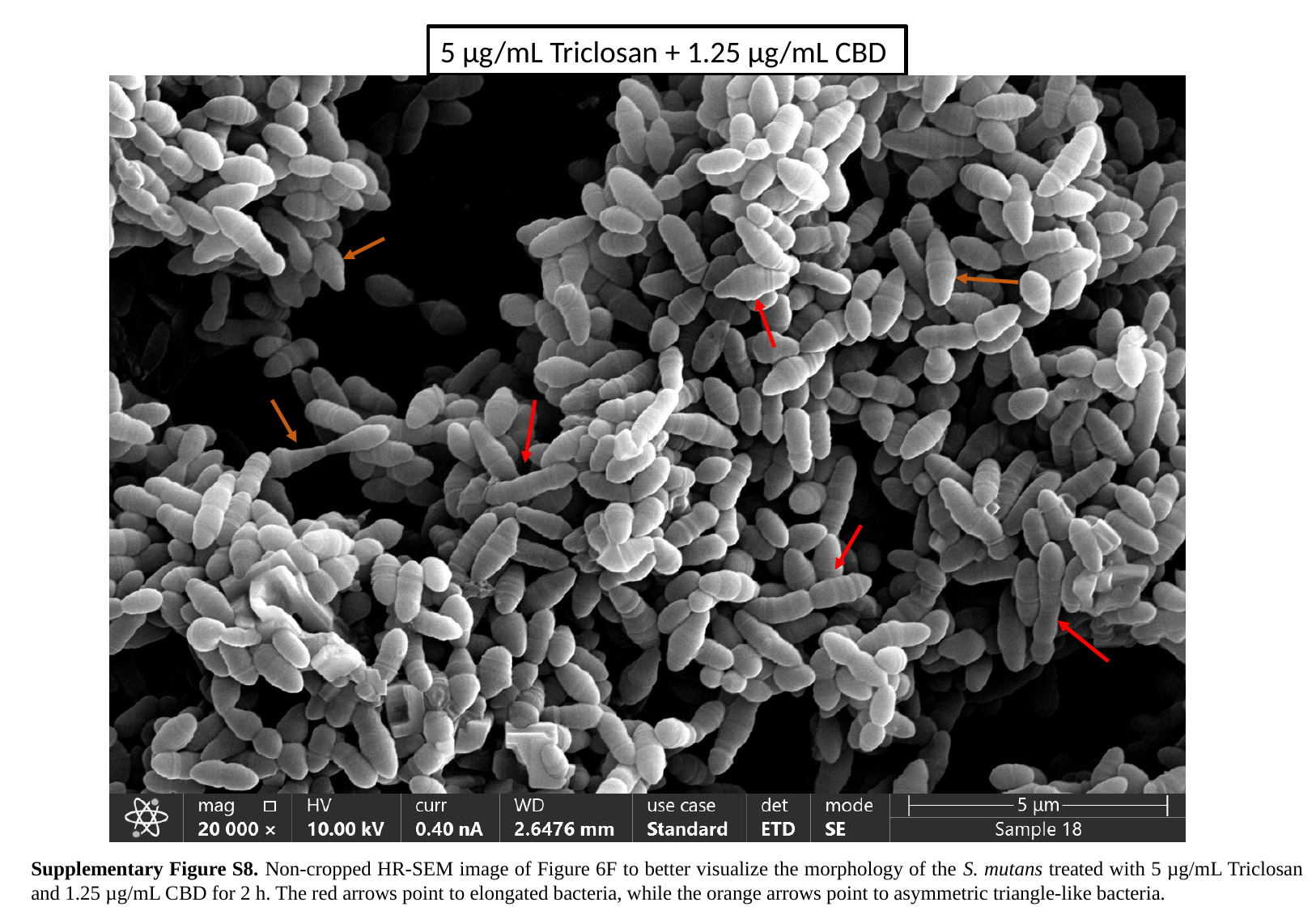

5 µg/mL Triclosan + 1.25 µg/mL CBD
Supplementary Figure S8. Non-cropped HR-SEM image of Figure 6F to better visualize the morphology of the S. mutans treated with 5 µg/mL Triclosan and 1.25 µg/mL CBD for 2 h. The red arrows point to elongated bacteria, while the orange arrows point to asymmetric triangle-like bacteria.

## Slide 9
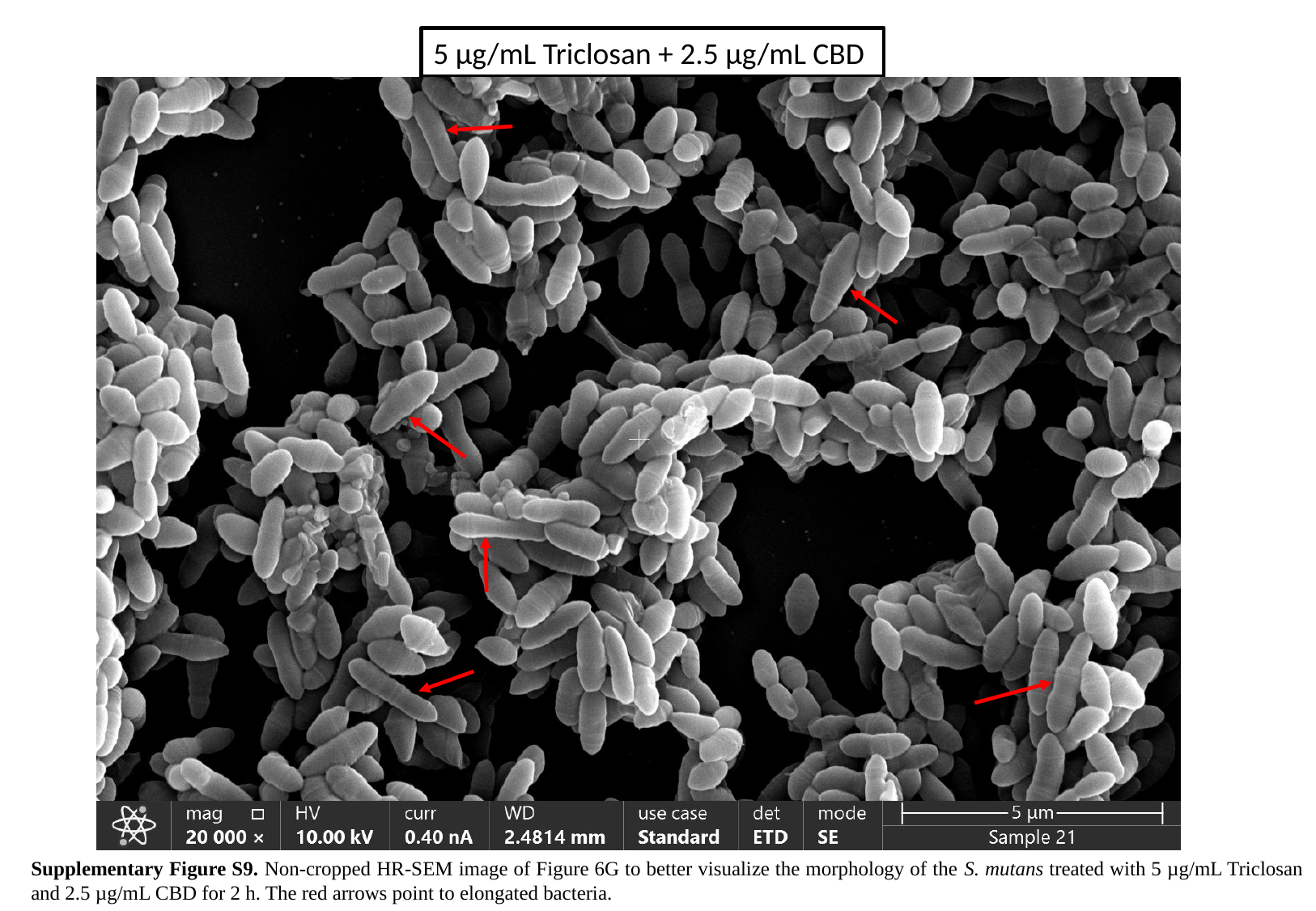

5 µg/mL Triclosan + 2.5 µg/mL CBD
Supplementary Figure S9. Non-cropped HR-SEM image of Figure 6G to better visualize the morphology of the S. mutans treated with 5 µg/mL Triclosan and 2.5 µg/mL CBD for 2 h. The red arrows point to elongated bacteria.

## Slide 10
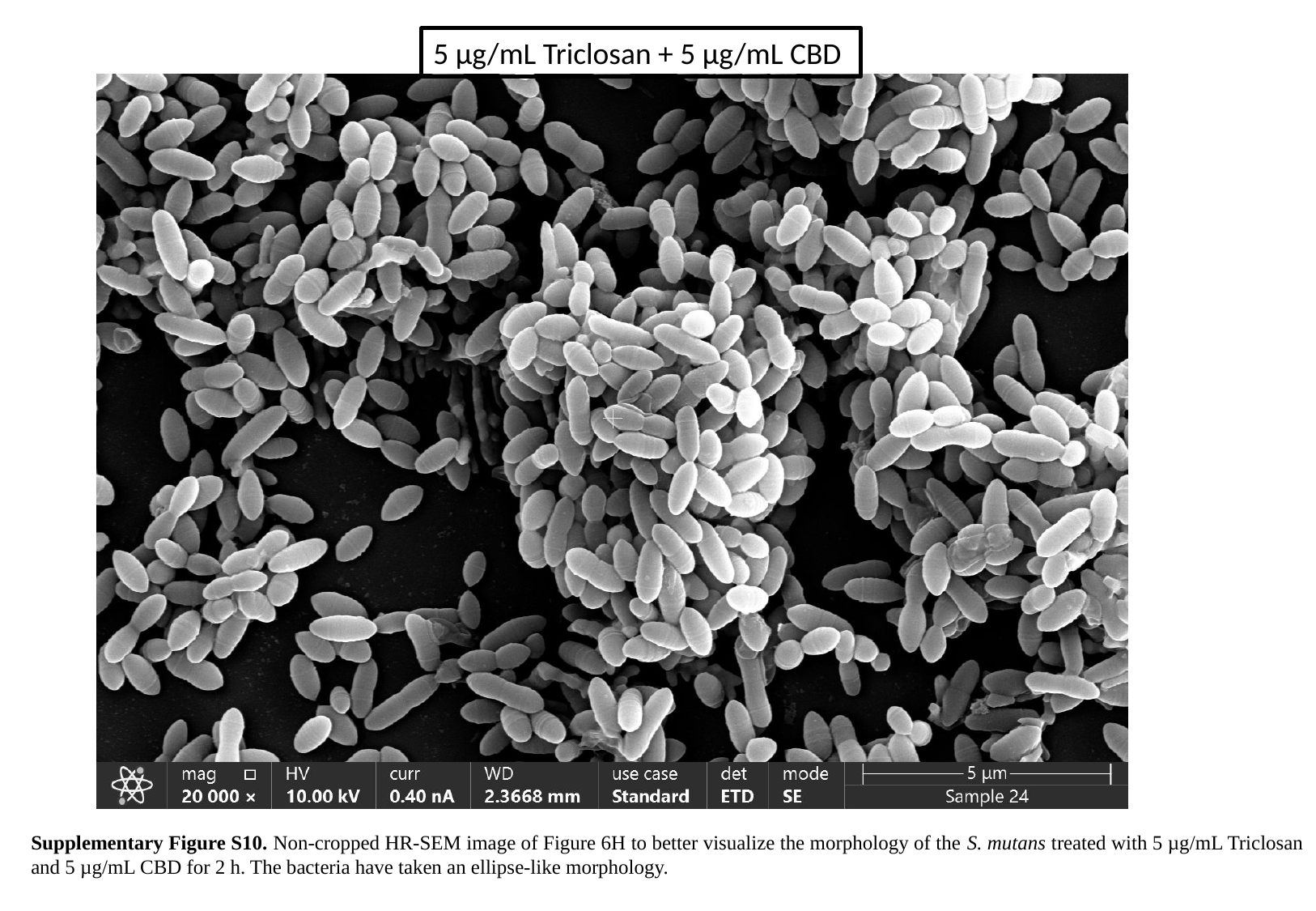

5 µg/mL Triclosan + 5 µg/mL CBD
Supplementary Figure S10. Non-cropped HR-SEM image of Figure 6H to better visualize the morphology of the S. mutans treated with 5 µg/mL Triclosan and 5 µg/mL CBD for 2 h. The bacteria have taken an ellipse-like morphology.

## Slide 11
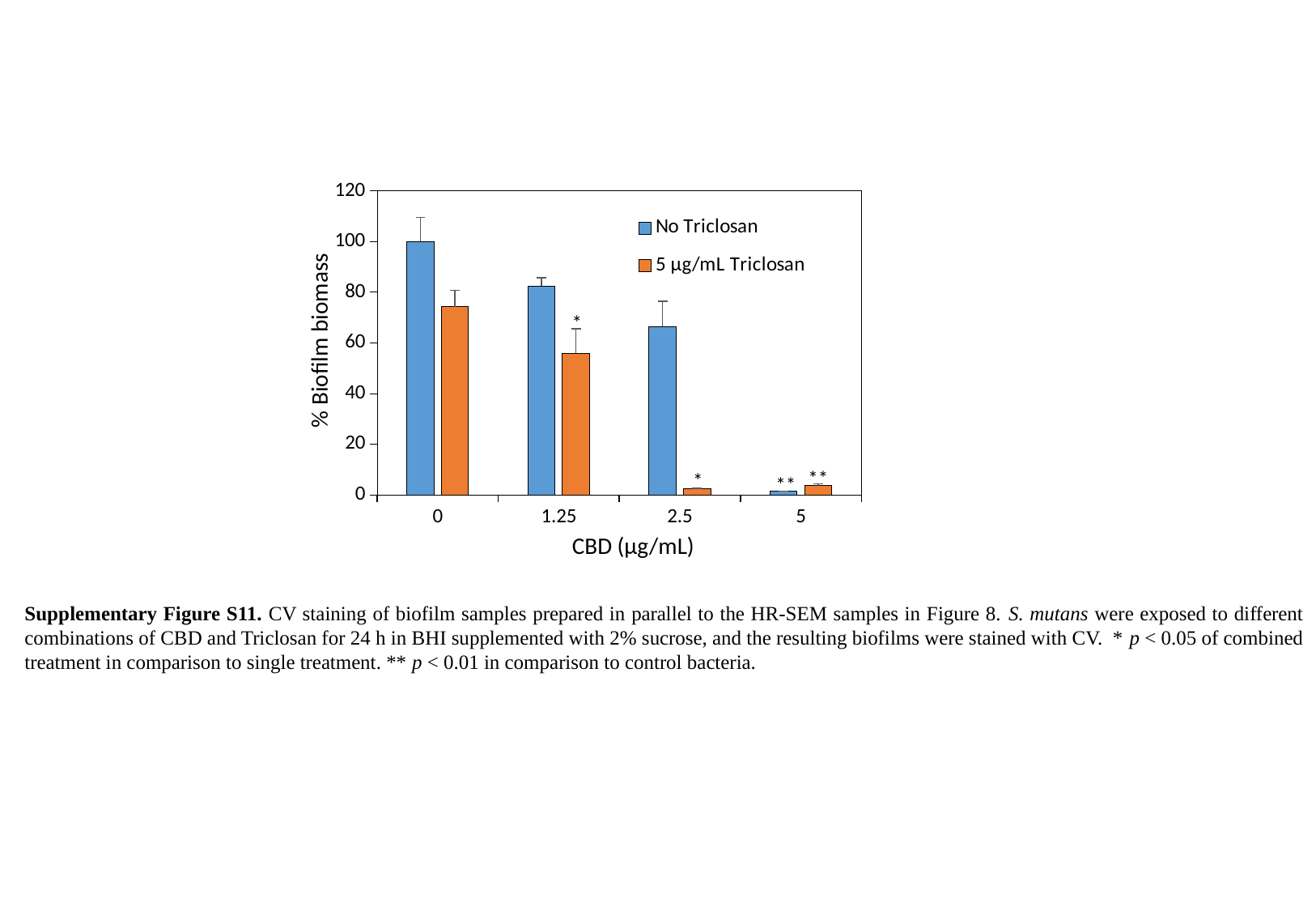

### Chart
| Category | No Triclosan | 5 µg/mL Triclosan |
|---|---|---|
| 0 | 100.0 | 74.3628466395124 |
| 1.25 | 82.48338723456621 | 56.0064280565271 |
| 2.5 | 66.44921096020218 | 2.614347328393596 |
| 5 | 1.482437071431921 | 3.7438233287959264 |*
% Biofilm biomass
**
*
**
CBD (µg/mL)
Supplementary Figure S11. CV staining of biofilm samples prepared in parallel to the HR-SEM samples in Figure 8. S. mutans were exposed to different combinations of CBD and Triclosan for 24 h in BHI supplemented with 2% sucrose, and the resulting biofilms were stained with CV. * p < 0.05 of combined treatment in comparison to single treatment. ** p < 0.01 in comparison to control bacteria.

## Slide 12
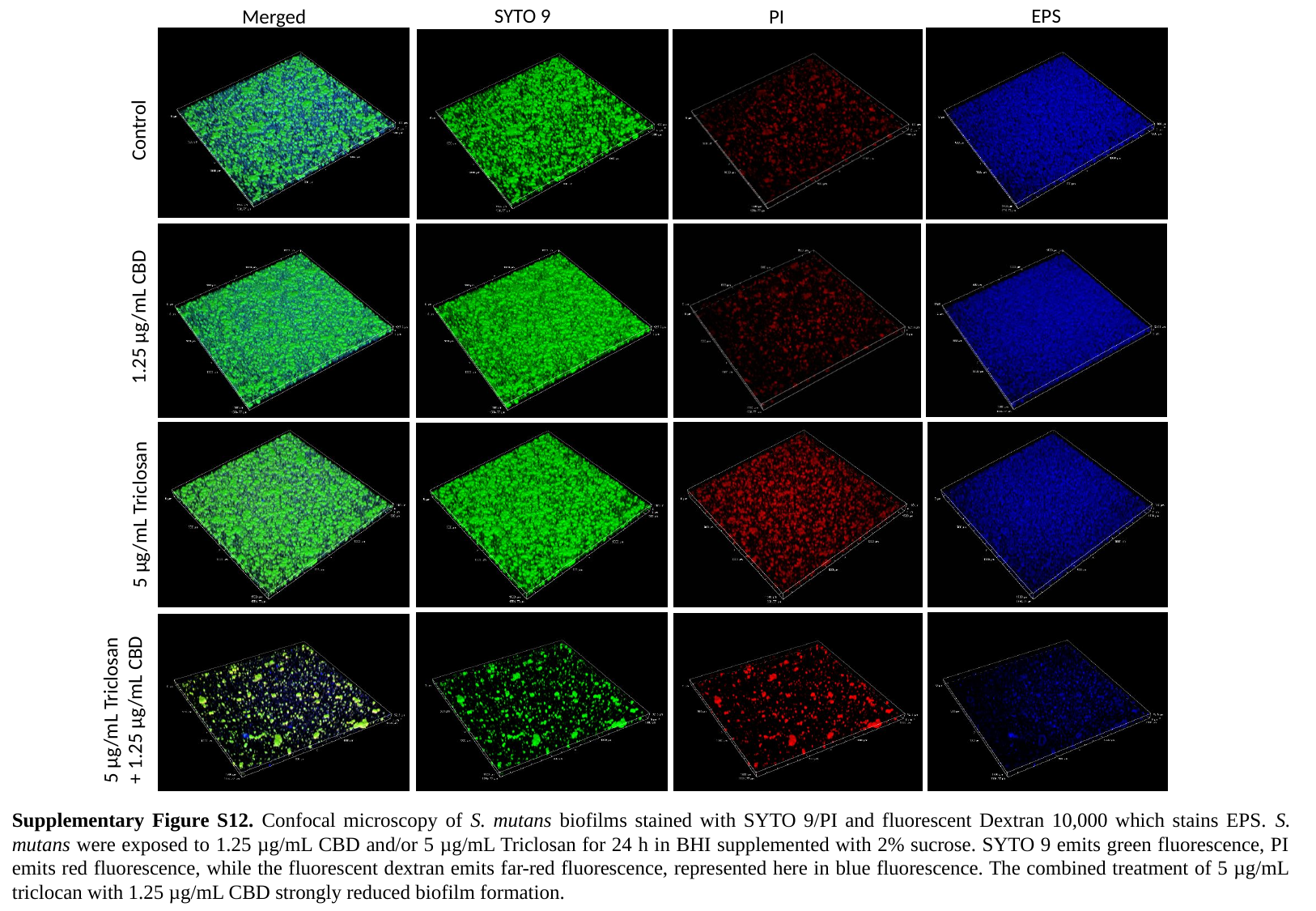

SYTO 9
EPS
Merged
PI
Control
1.25 µg/mL CBD
5 µg/mL Triclosan
5 µg/mL Triclosan
+ 1.25 µg/mL CBD
Supplementary Figure S12. Confocal microscopy of S. mutans biofilms stained with SYTO 9/PI and fluorescent Dextran 10,000 which stains EPS. S. mutans were exposed to 1.25 µg/mL CBD and/or 5 µg/mL Triclosan for 24 h in BHI supplemented with 2% sucrose. SYTO 9 emits green fluorescence, PI emits red fluorescence, while the fluorescent dextran emits far-red fluorescence, represented here in blue fluorescence. The combined treatment of 5 µg/mL triclocan with 1.25 µg/mL CBD strongly reduced biofilm formation.

## Slide 13
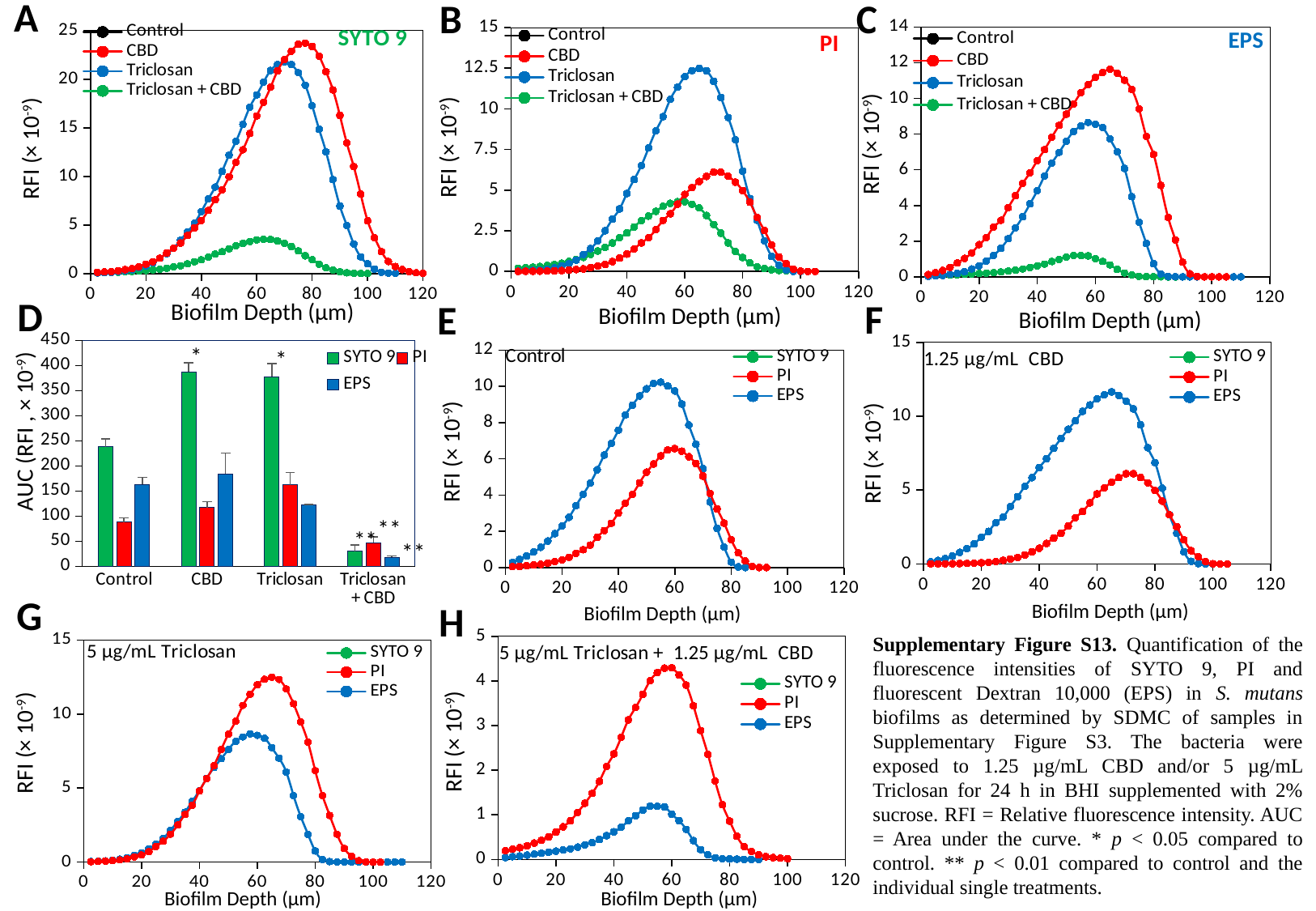

A
B
C
### Chart: SYTO 9
| Category | Control | CBD | Triclosan | Triclosan + CBD |
|---|---|---|---|---|
### Chart: PI
| Category | Control | CBD | Triclosan | Triclosan + CBD |
|---|---|---|---|---|
### Chart: EPS
| Category | Control | CBD | Triclosan | Triclosan + CBD |
|---|---|---|---|---|RFI (× 10-9)
RFI (× 10-9)
RFI (× 10-9)
D
F
E
Biofilm Depth (µm)
Biofilm Depth (µm)
Biofilm Depth (µm)
### Chart: Control
| Category | | | |
|---|---|---|---|
### Chart
| Category | SYTO 9 | PI | EPS |
|---|---|---|---|
| Control | 237.679183526 | 87.54272581959998 | 162.6309636734 |
| CBD | 387.02589402949997 | 116.4026178065 | 184.03482455600002 |
| Triclosan | 377.343093838 | 161.79764916749997 | 122.27619563350001 |
| Triclosan + CBD | 29.8572092875 | 46.548690158999996 | 17.6441348555 |
### Chart: 1.25 µg/mL CBD
| Category | | | |
|---|---|---|---|*
*
AUC (RFI , × 10-9)
RFI (× 10-9)
RFI (× 10-9)
**
**
**
G
H
Biofilm Depth (µm)
Biofilm Depth (µm)
### Chart: 5 µg/mL Triclosan
| Category | | | |
|---|---|---|---|
### Chart: 5 µg/mL Triclosan + 1.25 µg/mL CBD
| Category | | | |
|---|---|---|---|Supplementary Figure S13. Quantification of the fluorescence intensities of SYTO 9, PI and fluorescent Dextran 10,000 (EPS) in S. mutans biofilms as determined by SDMC of samples in Supplementary Figure S3. The bacteria were exposed to 1.25 µg/mL CBD and/or 5 µg/mL Triclosan for 24 h in BHI supplemented with 2% sucrose. RFI = Relative fluorescence intensity. AUC = Area under the curve. * p < 0.05 compared to control. ** p < 0.01 compared to control and the individual single treatments.
RFI (× 10-9)
RFI (× 10-9)
Biofilm Depth (µm)
Biofilm Depth (µm)
